# Full Recovery from O’Donoghue’s Triad with Autologous Bone Marrow Aspirate Matrix: A Case Report

**DOI:** 10.3390/jfmk7040100

**Published:** 2022-11-11

**Authors:** José Fábio Santos Duarte Lana, André Atsushi Sugano, Henrique Valadão De Barros, Tomas Mosaner, Gabriel Silva Santos, João Vitor Bizinotto Lana, Rodrigo Vicente, Marco Antônio Percope De Andrade

**Affiliations:** 1 Department of Orthopedics, Brazilian Institute of Regenerative Medicine, Indaiatuba 13334-170, Brazil; 2 Department of Orthopedics, SAMAX—Saúde Máxima, São Paulo 01239-040, Brazil; 3 Department of Locomotor Apparatus, The Federal University of Minas Gerais, Belo Horizonte 31270-901, Brazil

**Keywords:** case report, knee injury, anterior cruciate ligament, orthobiologics, bone marrow aspirate

## Abstract

O’Donoghue’s triad is an extremely debilitating condition. Although there are many conventional treatments available, there is still no consensus regarding the most effective rehabilitation protocol for a full recovery. Surgical interventions have become an ordinary consideration, but problems may still persist even after the surgical procedure. Orthobiologics, however, have gained considerable popularity in regenerative medicine. Notable autologous alternatives, such as bone marrow aspirate (BMA), are often utilized in clinical settings. To our knowledge, the administration of BMA products for the management of O’Donoghue’s triad has not been thoroughly investigated in the literature. In this case report we describe a full recovery from O’Donoghue’s triad with BMA matrix in a patient who was recalcitrant to surgical intervention due to fear of complications. Our patient received three BMA matrix injections with four-week intervals, exhibiting significant recovery according to pain scores, functional assessment outcomes, and magnetic resonance imaging (MRI) results. The patient returned to normal activities with no complaints and MRI evidence at follow-up showed significant signs of structural restoration of the musculoskeletal tissues. Here, we demonstrate that autologous BMA products are a feasible alternative for the accelerated recovery of musculoskeletal tissue injury with safety and efficacy.

## 1. Introduction

ACL ruptures can occur in contact sports and also in noncontact situations during sudden direction changes, cutting maneuvers, or during landing after a jump. Few prospective studies have investigated the biomechanical risk factors of ACL injuries, but it seems that the injury is linked to poor neuromuscular control of the knee-stabilizing muscles and to the dynamic valgus condition to which the knee can be subjected even in the context of contact sports [1]. This severe injury usually affects proximal structures, including the menisci, surrounding musculature, critical neurovascular structures, and other ligaments [2]. Moreover, it is also related to a higher risk of a knee re-injury and long-term medical disability due to early osteoarthritis occurring in half of the individuals 10–15 years later [3]. The ACL is a pivotal structure in knee joints and its main function is to avoid anterior translation of the tibia. It also stabilizes internal tibial rotation and valgus angulation at the knee [2]. Upon complete extension, the ACL can absorb up to 75% of loading and approximately 85% between 30 and 90 degrees of flexion [2]. ACL injuries promote biomechanical instability and reduced magnitude of coupled rotation during flexion. For reference, the tensile strength of this ligament is of about 2200 Newtons; however, this threshold may change due to advanced age and repetitive loading. ACL force increases in equal proportion to the increasing magnitude of the anterior drawer force [4].

Diagnosis and treatment can sometimes prove to be challenging and controversial, which is why a solid understanding of the knee joint and its biomechanics is required. This allows physicians to detect findings with more accuracy and design the most suitable approach with regard to reconstructive surgery or nonoperative alternatives, adding value to the care of individuals with soft tissue injuries. Regardless of circumstance, patients with ACL injuries are much more susceptible to additional problems [5]. A weakening of the quadriceps and hamstrings muscle groups, for example, is a common consequence of ACL injury and reconstruction, and this debilitation persists well beyond the post-operative rehabilitation period [5]. 

Although ligament reconstruction procedures are known to improve knee stability in ACL-deficient knees, they do not restore regular knee kinematics [6]. In fact, it usually facilitates the development of post-traumatic OA. Once this ligament is torn, a cascade of pathogenic processes is initiated, leading to increased turnover of aggrecan and Type II collagen in a matter of days [7]. This lies in parallel with the reports that were published by Nelson et al., with the observations of early post-traumatic OA-like alterations in human articular cartilage one year after ACL rupture [8]. 

With the continuous advances in regenerative medicine, practitioners have been able to appreciate the enhanced biological potential of orthobiologics. These products are derived from biological materials that are naturally found in the body and have the potential to accelerate healing of orthopedic conditions [9]. Notable alternatives such as platelet-rich plasma (PRP), hyaluronic acid (HA), and bone marrow aspirate/concentrate (BMA/BMAC) are often utilized in clinical settings [10].

BMA can be considered a feasible orthobiologic tool due to its remarkable regenerative attributes, even more so with regards to the treatment of musculoskeletal conditions [9]. BMA contains two major types of adult stem cells: mesenchymal stem cells (MSCs) and hematopoietic stem cells (HSCs) [10]. Both are renowned for the capacity to produce a wide array of bioactive molecules which allow them to manipulate the local tissue microenvironment via paracrine and autocrine signaling [10]. They are also praised for their significant self-renewal capacity and ability to differentiate into specific mature cell lineages, further enhancing tissue repair [10]. The administration of orthobiologics for the management of ACL injuries has not been extensively discussed in the literature, including cases of O’Donoghue’s triad.

Here, we discuss the application of the “BMA matrix” [10] as a feasible orthobiologic alternative for O’Donoghue’s triad.

## 2. Patient Information

**Figure 1:**
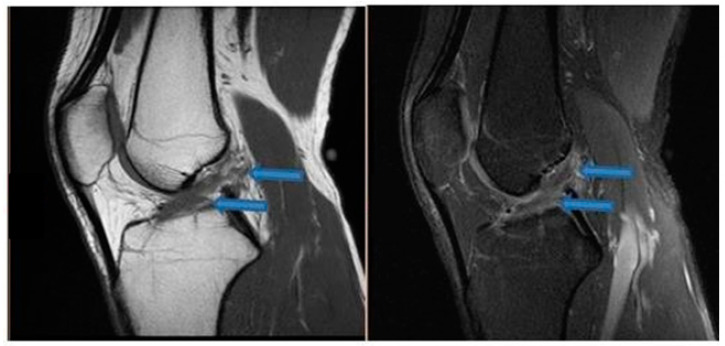
Sagittal T1- and T2-weighted MRI of the knee showing partial healing of the ACL indicated by blue arrows (9 November 2020).

**Figure 2:**
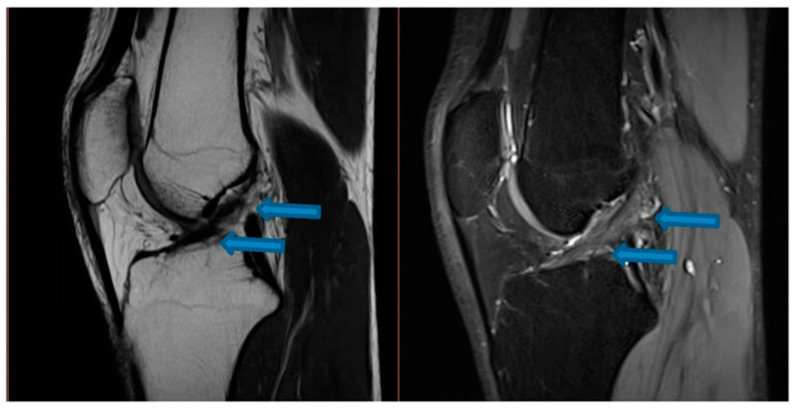
Sagittal T1- and T2-weighted MRI of the knee demonstrating signs of complete ACL healing indicated by blue arrows (14 August 2021).

A 30-year-old man arrived at the emergency department after twisting the right knee during a soccer match (20 July 2020). He exhibited intense pain (6 on the VAS scale) and Grade IV knee edema, being unable to walk, fully flex, or extend the knee. An immediate physical examination and MRI scans revealed ACL injury that was associated with MCL and medial meniscus injury (Figure 1 and Figure 2), leading to the characterization of “O’Donoghue’s triad”. Although our patient was in a good health state with no history of previous orthopedic injuries, he was recalcitrant to operative treatment for personal reasons and, therefore, sought orthobiologic treatment at our clinic.

## 3. Clinical Findings

**Figure 3:**
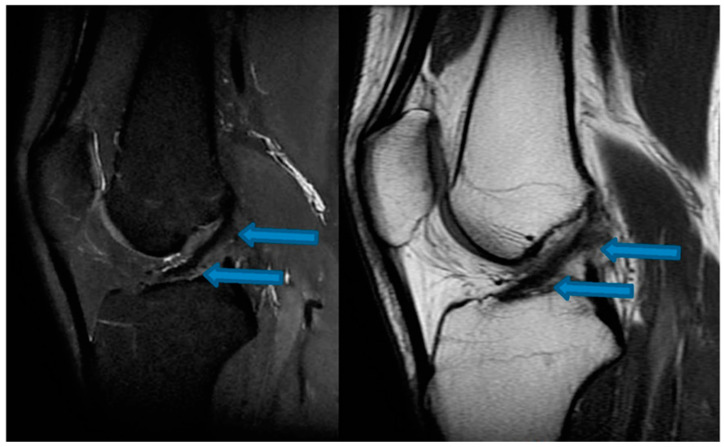
Sagittal T1- and T2-weighted MRI of the knee confirming complete healing of the ACL indicated by blue arrows (24 December 2021).

**Figure 4:**
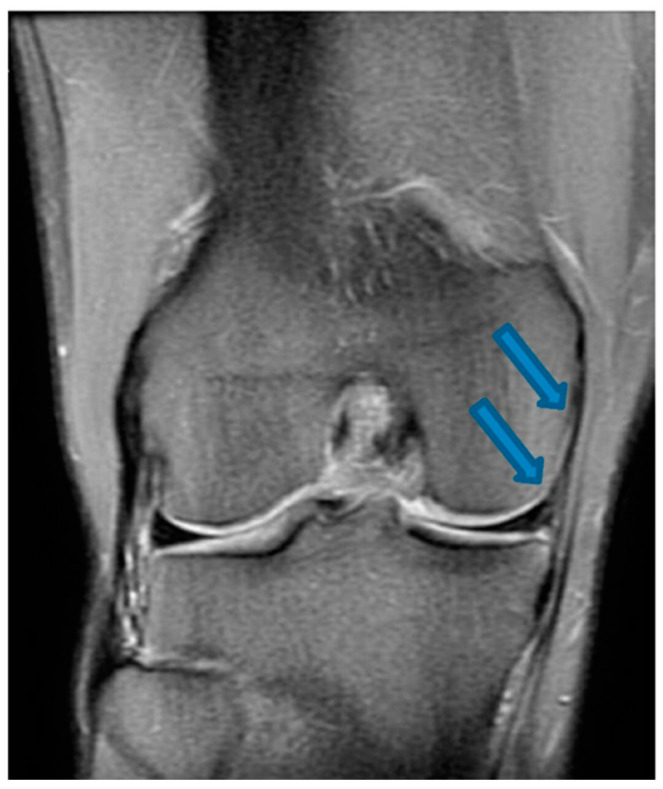
Coronal T2-weighted MRI of the knee showing complete MCL and medial meniscus healing indicated by blue arrows (24 December 2021).

One month after the first BMA matrix injection, the patient returned to our office for another physical examination. The individual reported an 80% improvement of symptoms without clinical instability, presenting a score of 2 on the VAS pain scale and an IKDC score of 74.7%. The patient received two additional BMA matrix injections with the 4-week intervals. MRI scans that were taken in November 2020 revealed clear signs of healing and elongation of the anterior cruciate ligament fibers with 45 degrees disposition (Figure 1). By the end of the treatment there was a significant alleviation of pain and an improvement of knee function in terms of the range of motion. Subsequent MRI results (Figure 2, Figure 3 and Figure 4) showed structural integrity and evidence of regeneration without any signs of recurrent injuries.

## 4. Timeline


**Date**

**Event**
20 July 2020Injury28 July 2020First BMA Matrix Injection26 August 2020Second BMA Matrix Injection25 September 2020Third BMA Matrix Injection9 November 2020MRI scans demonstrating signs of partial ACL healing14 August 2021MRI scans demonstrating signs of complete ACL healing24 December 2021MRI scans confirming signs of complete ACL, MCL and medial meniscus healing

## 5. Diagnostic Assessment

The IKDC (Table 1) and VAS scores (Table 2) were utilized in order to evaluate the severity of the patient’s physical condition. At rest, the VAS pain scores were 3 whereas scores for pain during movement were a 7. 

**Figure 5:**
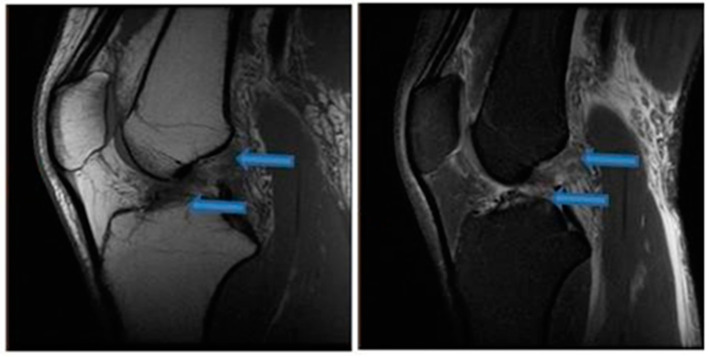
Sagittal T1- and T2-weighted magnetic resonance imaging of the knee demonstrating ACL detachment at the femoral origin indicated by blue arrows (20 July 2020).

**Figure 6:**
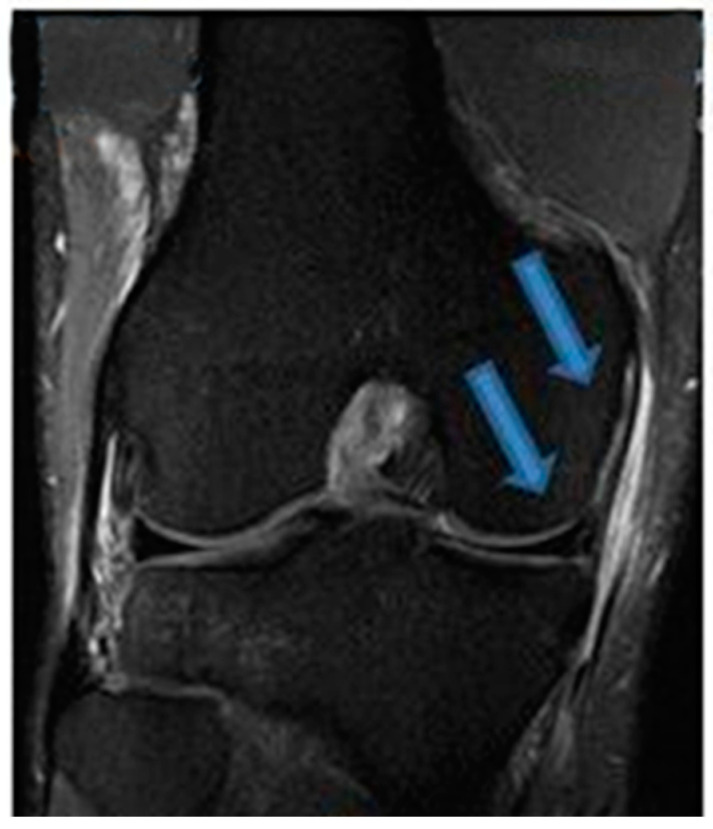
Coronal T2-weighted magnetic resonance imaging of the knee demonstrating injury of the medial meniscus and MCL indicated by blue arrows (20 July 2020).

Our patient exhibited intense pain (6 on the VAS scale) and Grade IV knee edema, being unable to walk, fully flex, or extend the knee. Immediate physical examination and MRI scans revealed ACL injury associated with MCL and medial meniscus injury (Figure 5 and Figure 6), leading to the characterization of “O’Donoghue’s triad”.

## 6. Therapeutic Intervention

On 28 July 2020, the patient received one BMA injection. Utilizing 10 mL syringes, 30 mL of fresh bone marrow was aspirated from the right posterior iliac crest. The syringe was repositioned after every 5 mL, allowing penetration of the deeper layers of bone to collect more stromal cells [11,12]. The product was then intra-articularly injected with ultrasound guidance. This protocol was repeated for the other sessions (Table 3). We recommended the patient avoid strenuous exercise and obtain sufficient rest; however, he was given permission to walk with physical aids and engage in low impact activities such as hydrotherapy and resistance exercise in order to further assist with strengthening and recovery of the injured tissues. 

## 7. Follow-Up and Outcomes

After a total of 30 days post-treatment, the patient reported an 80% improvement of symptoms. By the 45th day, the patient reported a remarkable gain in knee range of motion and progressive attenuation of pain. By the 60th day, the patient was asymptomatic and able to return to physical activities. No adverse events were reported during the intervention and follow-up except for the expected minor swelling after the injections. Table 1 and Table 2 speak directly to our observations regarding improvement of symptoms that were reported by the patient’s own perception and our clinical evaluation via physical assessments. MRI scans that were conducted from November 2020 to December 2021 revealed progressive signs of tissue healing (Figure 2, Figure 3 and Figure 4), further confirming our observations and expectations.

## 8. Discussion

O’Donoghue’s triad is a highly debilitating condition that causes major pain and significant decreases in an individual’s quality of life [13]. The incidence of these injuries among young sportsmen has increased significantly in the last two decades, yet there is almost no consensus regarding the most effective rehabilitation protocols for the achievement of complete physical recovery. Surgeries have become an ordinary alternative for athletes, whereas the initial conservative strategies concerning physical therapy are usually applied in the general population [14]. This creates additional challenges including prolonged pain, reduced physical capacity, and financial burden [7]. Weakness in the distal lower extremities is a common consequence that is attributed to ACL injury and reconstruction; it often persists even after the post-operative rehabilitation period [5]. Ligament reconstruction is known to improve knee stability in ACL-deficient knees; however, it does not restore regular knee kinematics. This can, in many cases, increase the risk of post-traumatic OA development [8]. ACL disruption initiates a cascade of pathogenic processes, including elevated turnover of aggrecan and Type II collagen in a matter of days [7]. 

Previous studies have reported the development of early post-traumatic OA-like alterations in human articular cartilage one year after ACL rupture [8]. Indeed, knee structures are major targets of shear forces throughout life, which is why physicians must be able to have a solid comprehension of knee anatomy and biomechanics in order to accurately identify and report the imaging findings. This optimizes decision-making in order to design the most suitable approach when it comes to reconstructive surgery or nonoperative alternatives.

In healthy patients, soft tissue injuries usually do not demand extended amounts of time to show signs of healing, but this also may vary according to injury severity. Our patient was only 30-years old when he suffered the accident and, based on the MRI scans and information that was collected via questionnaires, he exhibited a speedy recovery with only three BMA matrix injections. As previously discussed, the ordinary strategies for the management of ACL injuries typically include surgical interventions aiming at ACL reconstruction [14]. However, given the fact that this strategy does not restore regular knee kinematics, it may, therefore, be necessary to employ novel alternatives such as the application of orthobiologics in order to accelerate tissue repair [10]. Bone marrow-derived products, for instance, are being widely applied in numerous diseases and have shown great promise as a powerful regenerative medicine tool so far, not only for musculoskeletal tissues but other organ systems as well [15]. 

Numerous studies discuss the administration of orthobiologics, including autologous bone marrow-derived products for soft tissue injuries, especially in the knee. However, the majority of studies either focus more on the bone compartment itself or more generalized diseases such as OA. The bone marrow carries a wide array of cellular and molecular components, especially the bioactive molecules that are released by resident cells and platelets [10]. In the BMA matrix, these components are retained in the fibrin matrix that is formed in virtue of the coagulation properties of BMA itself, triggering regenerative mechanisms which are relatively similar to fracture hematomas [10]. We make use of the term “BMA matrix” in reference to our previously published material, proposing refinement of BMA application protocols [10]. This product is obtained via the intentional activation of the coagulation cascade with the typical cross-linking of fibrin fibers, thus forming a natural autologous matrix. This fibrin matrix acts as a biological scaffold and reservoir of cytokines and growth factors, facilitating MSC activity [10]. As mentioned in the introduction, MSCs are able to perform self-renewal and differentiate into mesodermal lineage cells, such as bone, fat, cartilage, muscle, meniscus, and tendons [10]. They also orchestrate autocrine and paracrine effects in order to manipulate the local tissue microenvironment. 

MSCs may offer major advantages as they are not known to trigger aggressive immunogenic responses and can be easily isolated, allowing allogenic transplantation procedures, if required [10]. However, the efficacy of such cellular-based therapies appears to be mostly linked to their ability of homing and engraftment into target tissues. MSCs have a comparably short life span and are eventually taken up by monocytes in order to stimulate the production of T-regulatory cells. This sequence of events significantly contributes to the overall clinical improvement [10]. 

Immediately after tissue injury events, local cells produce cytokines in order to recruit MSCs. Once recruited, these cells respond by producing molecular agents such as vasculoendothelial growth factor (VEGF), transforming growth factor beta (TGF-β), stromal-derived factor 1 (SDF-1), and stem cell factor (SCF), for example. Such growth factors allow MSCs to further regulate healing by halting fibrosis and apoptosis, attenuating escalated inflammation, and stimulating proliferation and differentiation of cells via paracrine and autocrine signaling [10]. 

The roles of interleukin-1 receptor antagonist (IL-1Ra) have also been documented. This potent cytokine that is found in the bone marrow acts as a competitive antagonist, binding to IL-1β and IL-1α receptors in order to impede IL-1-induced catabolic reactions and inflammatory stress [10]. This may partially explain why our patient exhibited significant improvements with regards to pain. IL-1Ra can decelerate the rate of matrix degradation, considering the fact that IL-1β induces the expression of matrix metalloproteinase (MMP) 3, tumor necrosis factor (TNF) alpha, and prostaglandin E2 (PGE2). It is also associated with chondrocyte apoptosis and the inhibition of collagen deposition [10]. In the case of ligament injuries, specifically the benevolent effects that are mediated by IL-1Ra, prove to be essential in order to restore tissue homeostasis and allow the proper reconstruction of fibers. 

BMA may also work well in combination with other orthobiologics for the treatment of several musculoskeletal disorders such as bone defects, joint arthritis, osteonecrosis, ligament tears, as well as pseudoarthrosis [16,17]. Previous studies have evaluated the application of bone marrow-derived products in ACL injuries [18,19,20,21,22,23]. A recent randomized controlled trial that was published by Centeno et al. [18] aimed at the evaluation of ACL tear healing with autologous bone marrow concentrate and platelets. The researchers concluded that the delivery of autologous BMC and platelets into ACL tears were able to significantly improve functional outcomes compared to exercise, observed through a follow-up period of 24-months. Individuals who received BMC showed quantitative improvements in post-treatment MRI scans, sustaining evidence of interval ligament healing. These findings are well aligned with our observations.

An animal study that was led by Oe et al. [19] demonstrated that the intra-articular delivery of fresh whole bone marrow cells (MSCs and HSCs) is an effective treatment alternative for a partial rupture of the ACL in rats. Undifferentiated MSCs release major growth factors and cytokines that trigger the expansion and differentiation of HSCs and regulate the response of immune cells during injury [20]. The authors reported notable effects, such as increased ligament strength, enhanced proliferation and migration of fibroblasts, elevated synthesis of collagen Type I fibers, and significant increases in PDGF and TGF-β1 expression [19]. These two growth factors stimulate ACL healing in vivo [21]. At least in in vitro settings, TGF-β1 has demonstrated the ability to accelerate the proliferation of ACL fibroblasts and extracellular matrix (ECM) synthesis [19]. This growth factor is not only secreted by fibroblasts but by many other cells types as well, including the ones that are differentiated from HSCs [19]. The researchers also reported that the inhibition of TGF-β1 activity is associated with negative effects, notably a significant decrease in tensile strength [19].

Mengsteab and colleagues [22] attempted to analyze the effects of biological factors and material composition on the regeneration and functional outcomes of a bioengineered ACL matrix. In their study, treatment with BMAC increased the expression of cuboidal cellular phenotypes that were similar to ACL fibroblasts and chondrocyte-like cells and enriched the ECM with anionic macromolecules. BMAC may serve as a rich reservoir of progenitor cells and trophic factors due to its relative abundance of growth factors and cytokines that contribute to angiogenesis, chondrogenesis, and MSC-homing [23]. They are essential for soft tissue repair, especially ligament tears. Additionally, fibroblast growth factor (FGF) 2 and 8 were found to significantly reduce the concentration of pro-inflammatory cytokines in synovial fluid. FGF-2, in particular, is known to be expressed in both MSCs and HSCs. It is an angiogenic and pleiotropic growth factor that is involved in the proliferation and differentiation of numerous cell types, playing a key role in mesoderm induction [24]. 

In this case report, our patient was very satisfied with the intervention and displayed a very positive response to treatment without serious adverse effects. One notable limitation of our study is that we were unable to employ additional methods of more objective evaluations other than being restricted to MRI and questionnaires. However, the wide success of orthobiologics in regenerative medicine still provided enough confidence to proceed with the administration of an autologous bone marrow-derived product, achieving the expected results. The BMA matrix promoted significant improvements in pain, functional outcomes, and expressive signs of soft tissue healing with only three sessions, with notable effects in just one month after a single infiltration. Although there are numerous studies evaluating bone marrow-derived products for general knee pathologies, there is still a significant lack of clinical investigations focusing on BMA for O’Donoghue’s triad specifically. Additional studies are highly warranted in order to better comprehend the mechanisms underpinning this technique. 

## Figures and Tables

**Table 1:** IKDC questionnaire scores.

Date	IKDC Questionnaire Scores
28 July 2020	21.8%
20 December 2020	74.7%
18 August 2021	96.6%
24 December 2021	100%

**Table 2:** VAS Scale scores.

Date	VAS Scale Scores
28 July 2020	7
20 December 2020	2
18 August 2021	2 (with maximum knee flexion)
24 December 2021	0

**Table 3:** Orthobiologic treatment design.

Date	Injection
28 July 2020	30 mL BMA (left posterior iliac crest)
26 August 2020	30 mL BMA (right posterior iliac crest)
25 September 2020	30 mL BMA (left posterior iliac crest)

## Data Availability

Not applicable.
